# Serine hydroxymethyl transferase 1 stimulates pro-oncogenic cytokine expression through sialic acid to promote ovarian cancer tumor growth and progression

**DOI:** 10.1038/onc.2017.37

**Published:** 2017-03-13

**Authors:** R Gupta, Q Yang, S K Dogra, N Wajapeyee

**Affiliations:** 1 Department of Pathology, Yale University School of Medicine, New Haven, CT, USA; 2 Singapore Institute of Clinical Sciences, Agency for Science Technology and Research (A*STAR), Brenner Center for Molecular Medicine, Singapore, Singapore

## Abstract

High-grade serous (HGS) ovarian cancer accounts for 90% of all ovarian cancer-related deaths. However, factors that drive HGS ovarian cancer tumor growth have not been fully elucidated. In particular, comprehensive analysis of the metabolic requirements of ovarian cancer tumor growth has not been performed. By analyzing The Cancer Genome Atlas mRNA expression data for HGS ovarian cancer patient samples, we observed that six enzymes of the folic acid metabolic pathway were overexpressed in HGS ovarian cancer samples compared with normal ovary samples. Systematic knockdown of all six genes using short hairpin RNAs (shRNAs) and follow-up functional studies demonstrated that serine hydroxymethyl transferase 1 (SHMT1) was necessary for ovarian cancer tumor growth and cell migration in culture and tumor formation in mice. *SHMT1* promoter analysis identified transcription factor Wilms tumor 1 (WT1) binding sites, and *WT1* knockdown resulted in reduced *SHMT1* transcription in ovarian cancer cells. Unbiased large-scale metabolomic analysis and transcriptome-wide mRNA expression profiling identified reduced levels of several metabolites of the amino sugar and nucleotide sugar metabolic pathways, including sialic acid *N*-acetylneuraminic acid (Neu5Ac), and downregulation of pro-oncogenic cytokines interleukin-6 and 8 (IL-6 and IL-8) as unexpected outcomes of SHMT1 loss. Overexpression of either IL-6 or IL-8 partially rescued SHMT1 loss-induced tumor growth inhibition and migration. Supplementation of culture medium with Neu5Ac stimulated expression of IL-6 and IL-8 and rescued the tumor growth and migratory phenotypes of ovarian cancer cells expressing *SHMT1* shRNAs. In agreement with the ovarian tumor-promoting role of Neu5Ac, treatment with Neu5Ac-targeting glycomimetic P-3Fax-Neu5Ac blocked ovarian cancer growth and migration. Collectively, these results demonstrate that SHMT1 controls the expression of pro-oncogenic inflammatory cytokines by regulating sialic acid Neu5Ac to promote ovarian cancer tumor growth and migration. Thus, targeting of SHMT1 and Neu5Ac represents a precision therapy opportunity for effective HGS ovarian cancer treatment.

## Introduction

According to the most recent estimates, ~22 280 new cases of ovarian cancer will be diagnosed and 14 240 women will die of this disease in the United States this year alone.^1^ Despite decades of research and with new treatment modalities, the 5-year survival rate of women with advanced (stage III and IV) ovarian cancer remains between 10 and 30%.^2^ Of these, high-grade serous (HGS) ovarian cancer accounts for 90% of all ovarian cancer-related deaths. Current therapies against advanced-stage ovarian cancer are largely ineffective, and thus effective and durable therapies are needed.

The Cancer Genome Atlas (TCGA) studies analyzed 489 HGS ovarian cancer samples for mRNA expression, microRNA expression, promoter DNA methylation and DNA copy number alterations. Additionally, 316 of these tumors were also analyzed by exome sequencing to identify mutations. Collectively, these studies identified recurrent mutations in 10 genes, focal copy number alterations in 113 genes and promoter methylation alterations of 168 genes, and captured transcriptional alterations in ovarian cancer samples compared with normal ovary samples.^3^ However, not many actionable alterations of therapeutic value for ovarian cancer patients have been identified thus far. This is, in part, due to the lack of functional studies to determine which of these alterations identified by the TCGA studies have decisive roles in promoting ovarian cancer tumor growth and progression.

Metabolic reprogramming of energetic and biosynthetic pathways is a hallmark of cancer cells.^4^ These metabolic changes allow cancer cells to proliferate even under conditions of limited nutrients and thus represent important vulnerabilities of therapeutic value. Although some studies have shown that metabolic alterations might have a role in ovarian cancer,^5, 6, 7^ the mechanisms underlying most metabolic deregulation in ovarian cancer remain to be identified. Previous studies, particularly in the context of metabolic syndrome, have shown that metabolic pathways can affect inflammation and inflammatory responses.^8, 9, 10^ However, the precise mechanisms by which these changes take place in cancer are not fully understood, and the role and implications of metabolic deregulation in the context of ovarian cancer have not been studied.

Here, by combining the TCGA ovarian cancer gene expression data set with the functional genomics approach of RNA interference, we demonstrated that the enzyme serine hydroxymethyl transferase 1 (SHMT1) is necessary for ovarian cancer tumor growth and progression. Surprisingly, we found that knockdown of *SHMT1* results in reduced levels of some metabolites, such as the sialic acid *N*-acetylneuraminic acid (Neu5Ac), in the amino sugar and nucleotide sugar metabolic pathway. We found that Neu5Ac stimulates the expression of the pro-oncogenic inflammatory cytokines interleukin-6 and -8 (IL-6 and IL-8), which was necessary for ovarian cancer tumor growth. Taken together, these findings identify SHMT1 as a new regulator of ovarian cancer tumor growth and progression that functions by increasing the expression of pro-oncogenic inflammatory cytokines through sialic acid Neu5Ac.

## Results

### SHMT1 is necessary for ovarian cancer tumor growth and cell migration

To identify metabolic alterations that are necessary for ovarian cancer tumor growth, we analyzed the TCGA gene expression data from 489 ovarian cancer samples and found that multiple genes encoding the enzymes for oxidative phosphorylation, histidine-glutamate-glutamine metabolism and folic acid metabolism were significantly upregulated in ovarian cancer samples compared with the normal ovary control (Supplementary Figure 1). The roles of oxidative phosphorylation and the histidine-glutamate-glutamine metabolic pathway have been previously documented in ovarian cancer.^11, 12^ Therefore, we focused our studies on the folic acid metabolism pathway.

**Figure 1:**
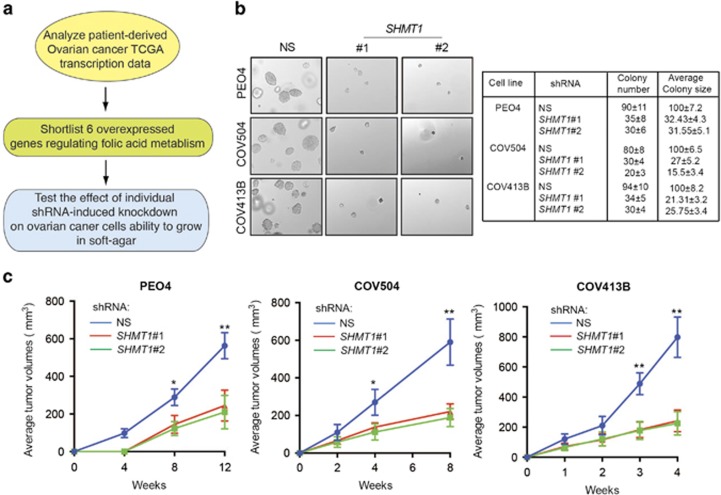
SHMT1 is necessary for ovarian cancer tumor growth. (**a**) Schematic diagram showing the functional validation pipeline for identifying the metabolic genes promoting ovarian cancer tumor growth. (**b**) The indicated ovarian cancer cell lines expressing control nonspecific (NS) or *SHMT1* shRNAs were analyzed for their ability to grow in an anchorage-independent manner in a soft-agar assay. Representative soft-agar assay images are shown (left) and relative colony numbers and average colony size are shown (right). (**c**) The indicated ovarian cancer cell lines expressing NS or *SHMT1* shRNAs were subcutaneously injected into the flanks of athymic nude mice and analyzed for tumor-forming ability. Average tumor volumes at indicated time points (*n*=5) are shown for ovarian cancer cell lines PEO4, COV504 and COV413B. Data are presented as mean±s.e.m.; *P<0.05 and **P<0.005.

In particular, we identified six genes encoding enzymes of folic acid metabolism that were significantly upregulated in ovarian cancer samples compared with normal ovary tissue (Supplementary Table 1 and Supplementary Figure 2). To determine whether any of these genes were necessary for ovarian cancer tumor growth, we knocked down each gene individually in the ovarian cancer cell line PEO4 using gene-specific short hairpin RNAs (shRNAs) (Supplementary Figure 3A). These cells were analyzed for the ability to grow in an anchorage-independent manner in a soft-agar assay (Figure 1a). The soft-agar assay is a surrogate assay to measure the tumorigenic potential of cancer cells and accurately mimics tumor growth *in vivo*.^13, 14^ Our results show that shRNA-induced knockdown of dihydrofolate reductase (*DHFR*), gamma-glutamyl hydrolase (*GGH*), phosphoribosylglycinamide formyltransferase (*GART/PUR2*), 5-aminoimidazole-4-carboxamide ribonucleotide formyltransferase/IMP cyclohydrolase (*ATIC/PUR9*) and serine hydroxymethyl transferase 2 (*SHMT2*) did not significantly affect the ability of PEO4 cells to form colonies in soft agar (Supplementary Figure 4). However, serine hydroxymethyl transferase 1 (*SHMT1*) knockdown was a potent inhibitor of PEO4 cell growth in soft agar (Figure 1b). Similarly, *SHMT1* knockdown inhibited the colony-forming ability of other ovarian cancer cell lines COV504 and COV413B in soft agar (Figure 1b and Supplementary Figure 3B).

To determine if loss of SHMT1 effects ovarian cancer tumor growth *in vivo*, we injected ovarian cancer cell lines expressing either shRNA targeting *SHMT1* or a nonspecific shRNA subcutaneously into the flanks of the athymic nude mice and measured tumor volume at specific time points after injection. In complete agreement with our soft-agar results, we found that loss of *SHMT1* inhibited ovarian tumor growth *in vivo* (Figure 1c).

**Figure 2:**
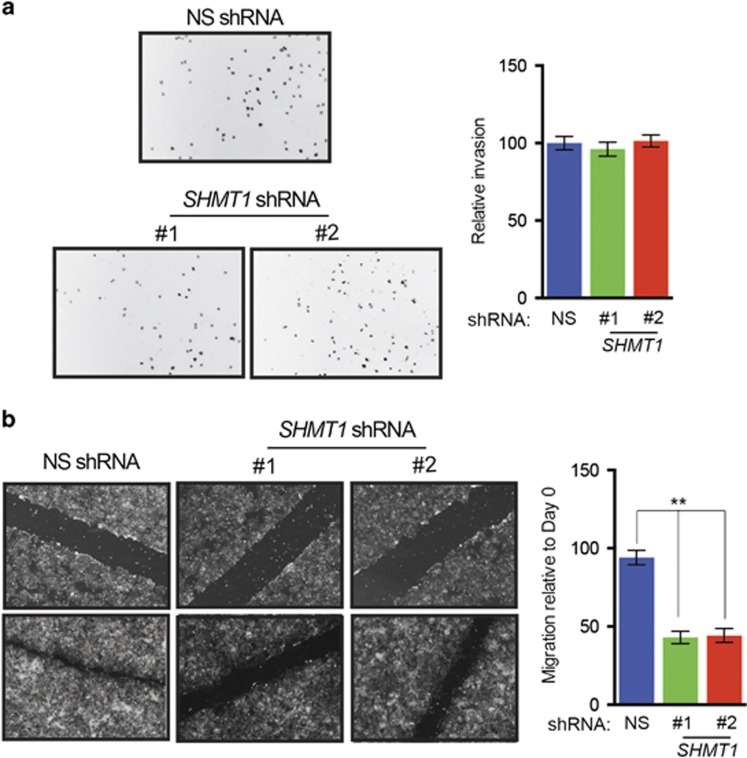
*SHMT1* knockdown inhibits ovarian cancer cell migration. (**a**) PEO4 cells expressing control nonspecific (NS) or *SHMT1* shRNAs were analyzed for invasive ability using Matrigel-based Boyden chamber assay. Representative images are shown (left) and quantitation is presented (right). (**b**) PEO4 cells expressing the indicated shRNAs were analyzed for migratory potential in a wound-healing assay. (Left) Representative images at days 0 and 4 are shown. (Right) Percent migration relative to day 0 is plotted. Data are presented as mean±s.e.m.; **P<0.005.

To determine if SHMT1 regulates phenotypes associated with tumor progression, we performed a wound-healing assay and Matrigel-based invasion assay. We found that *SHMT1* knockdown did not inhibit invasion of ovarian cancer cells (Figure 2a), but did result in significant reduction in the ability of ovarian cancer cells to migrate, as demonstrated by impaired wound healing in cells expressing *SHMT1* shRNAs compared with cells expressing nonspecific shRNAs (Figure 2b). In contrast, knockdown of other genes in the folic acid metabolic pathway did not significantly affect the migratory ability of ovarian cancer cells (Supplementary Figure 5).

### Transcription factor WT1 is necessary for transcriptional upregulation of *SHMT1*

**Figure 3:**
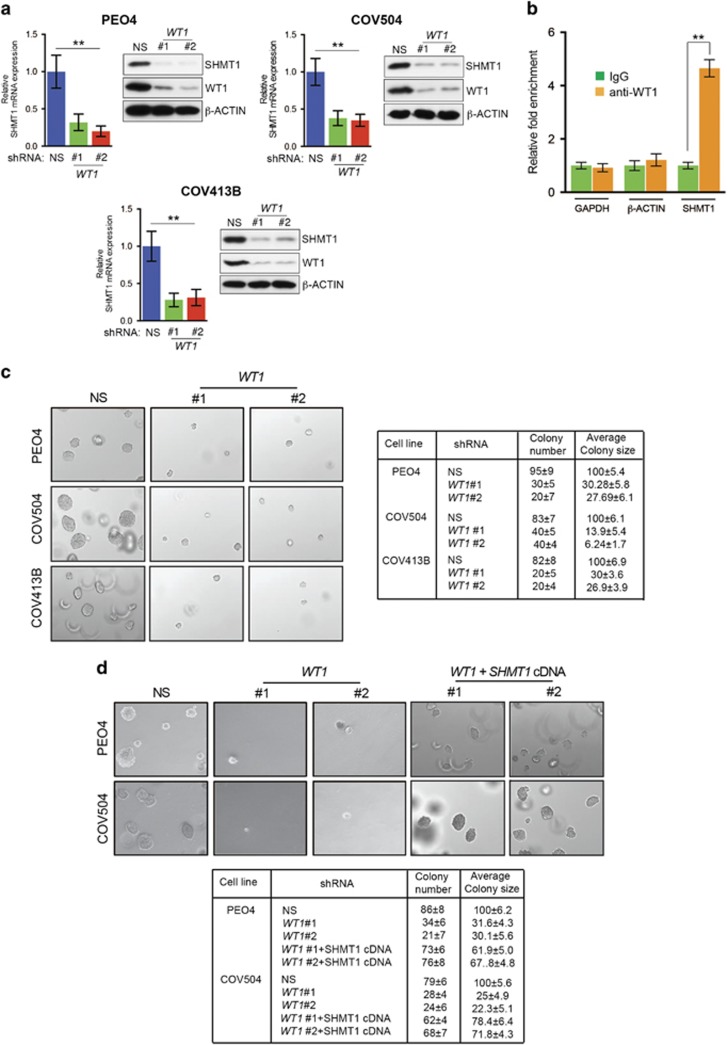
Transcription factor WT1 regulates *SHMT1* transcription in ovarian cancer cells. (**a**) RT–qPCR analysis of *SHMT1* mRNA expression and immunoblot analysis of WT1 and SHMT1 levels in the indicated ovarian cancer cell lines expressing either control nonspecific (NS) or *WT1* shRNAs. *SHMT1* mRNA level was normalized to that of β-ACTIN, and β-ACTIN served as a loading control on blots. (**b**) PEO4 cells expressing NS or *WT1* shRNAs were analyzed for WT1 protein enrichment on *SHMT1* promoter using chromatin immunoprecipitation assay. β-ACTIN and *GAPDH* promoter regions were used as negative controls. Percent WT1 enrichment relative to input for indicated conditions for each promoter locus (*SHMT1*, β-ACTIN and *GAPDH*) is shown. (**c**) PEO4, COV504, and COV413 cells expressing indicated NS or *WT1* shRNAs were analyzed for ability to grow in an anchorage-independent manner in a soft-agar assay. Representative soft-agar assay images are shown on left and relative colony numbers and average colony size are shown on right. (**d**) PEO4 cells expressing NS or *WT1* shRNAs with either an empty vector or *SHMT1* cDNA were analyzed for the ability to grow in an anchorage-independent manner in a soft-agar assay. Representative soft-agar assay images are shown on the left, and relative colony numbers and average colony size are shown on the right. Data are presented as mean±s.e.m.; **P<0.005.

Based on our analysis of the TCGA data set, *SHMT1* mRNA was upregulated in ovarian cancer samples (Supplementary Figure 2). To determine the mechanism by which *SHMT1* transcription is upregulated in these cells, we analyzed the *SHMT1* promoter sequence using the transcription factor DNA-binding site-prediction programs PROMO and rVISTA 2.0.^15, 16^ These analyses identified a conserved DNA-binding site for the transcription factor Wilms tumor 1 (WT1) (Supplementary Figure 6). WT1 exerts cancer-promoting activities in different types of cancers, and WT1 upregulation has been shown to correlate with poor prognosis in ovarian cancer patients.^17, 18^ Based on our analysis and these observations, we tested the role of WT1 in transcriptional upregulation of *SHMT1*. To this end, we knocked down *WT1* using shRNAs in three ovarian cancer cell lines (PEO4, COV504 and COV413B) (Figure 3a). We found that *WT1* knockdown in ovarian cancer cell lines resulted in reduced *SHMT1* expression (Figure 3a).

To determine if WT1 is a direct regulator of *SHMT1* transcription, we performed a chromatin immunoprecipitation assay to investigate association of WT1 with the *SHMT1* gene promoter sequence. Our results show that WT1 was significantly enriched on the *SHMT1* promoter sequence compared with the *ACTB* or *GAPDH* promoter sequences (Figure 3b).

To assess whether loss of WT1 results in inhibition of ovarian cancer cell colony formation, we performed a soft-agar assay. We found that, similar to *SHMT1* knockdown, *WT1* knockdown also prevented the ability of ovarian cancer cells to form colonies in soft agar (Figure 3c). To further confirm that WT1-mediated *SHMT1* upregulation has a key role in the ability of WT1 to promote ovarian cancer tumor growth, we transfected cells expressing *WT1* shRNA with constructs carrying *SHMT1* cDNA and measured growth in the soft-agar assay. We found that expression of exogenous *SHMT1* in *WT1* shRNA-expressing ovarian cancer cells rescued the cells' ability to form colonies in soft agar (Figure 3d). Collectively, these results demonstrate that WT1 is necessary for transcriptional upregulation of *SHMT1*, which mediates cancer-promoting activity of WT1.

### An unbiased large-scale metabolomic analysis revealed alterations in the amino sugar and nucleotide sugar metabolic pathway in *SHMT1* knockdown ovarian cancer cells

Because SHMT1 is a metabolic enzyme, we performed a large-scale unbiased metabolomic analysis with the goal of understanding the mechanism of SHMT1 action. To do so, PEO4 cells expressing control nonspecific shRNA or shRNA targeting *SHMT1* were analyzed using capillary electrophoresis time-of-flight mass spectrometry in two modes, for cationic and anionic metabolites. We detected 175 different metabolites of different metabolic pathways.

**Figure 4:**
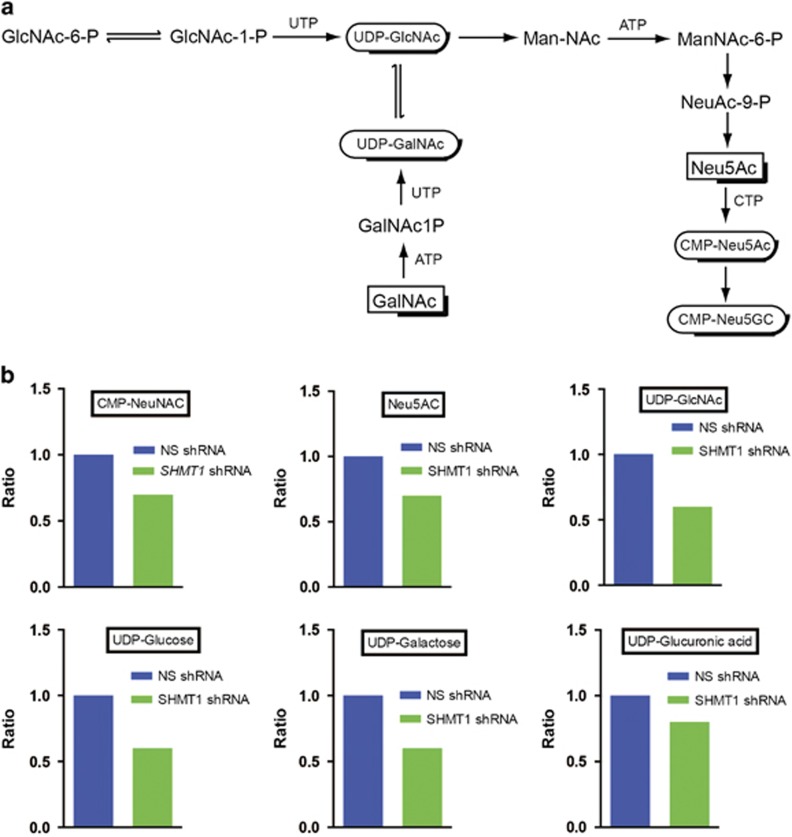
SHMT1 loss results in reduced amino sugar and nucleotide sugar metabolic pathway intermediates in ovarian cancer cells. (**a**) Key steps of the amino sugar and nucleotide sugar metabolic pathway. (**b**) Relative concentrations of indicated metabolites in *SHMT1* knockdown PEO4 cells compared with cells expressing nonspecific (NS) shRNA.

We found that multiple metabolites of the amino sugar and nucleotide sugar metabolic pathway (Figures 4a and b) were significantly downregulated upon *SHMT1* knockdown. In particular, knockdown of *SHMT1* resulted in downregulation of several intermediates of nucleotide sugar biosynthetic pathways, including CMP-*N*-acetylneuraminic acid, *N*-acetylneuraminic acid, UDP-*N*-acetylglucosamine, UDP-glucose, UPD-galactose and UDP-glucuronic acid (Figure 4b). Nucleotide sugars are needed to activate metabolites for various anabolic processes and include UDP-glucose and other UDP-hexoses (for carbohydrates synthesis), CDP-choline (for lipid synthesis), GDP sugars (e.g., GDP mannose for glycosyltransferases), NAD(P)+, FAD/FMN (for mediating redox reactions) and ADP ribose for a wide range of regulatory functions.^19^ In mammals, the nucleotide sugar biosynthetic pathway requires UDP-*N*-acetylglucosamine to produce Neu5Ac, a predominant sialic acid.^20^ Sialic acid sugars are overexpressed by cancer cells and contribute to tumor progression by affecting various aspects of tumor biology.^20, 21^ Our metabolomic analysis showed that *SHMT1* knockdown results in decreased Neu5Ac level in ovarian cancer cells (Figure 4b) and that Neu5Ac may have an important role in the regulation of ovarian cancer tumor growth and progression.

### Transcriptome-wide mRNA expression profiling shows reduced IL-6 and IL-8 levels in *SHMT1* knockdown ovarian cancer cells

**Figure 5:**
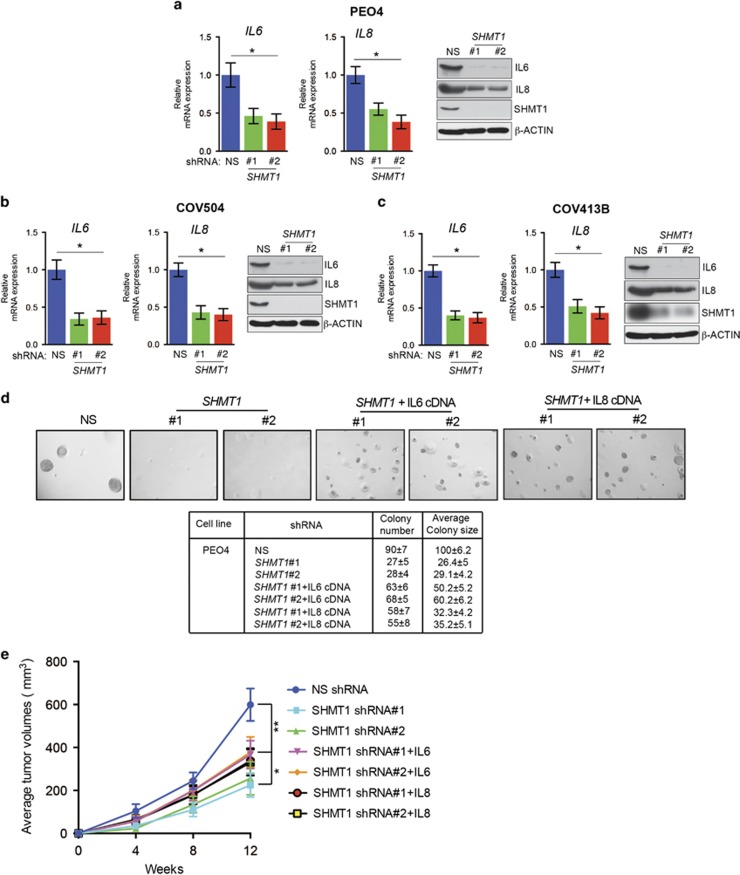
SHMT1 loss results in reduced levels of pro-oncogenic inflammatory cytokines that are necessary for SHMT1-mediated ovarian cancer growth. (**a**–**c**) PEO4 (**a**), COV504 (**b**) and COV413B (**c**) cells expressing control nonspecific (NS) or *SHMT1* shRNAs were analyzed for the expression of *IL-6* and *IL-8* mRNA by RT–qPCR (left) and of IL-6, IL-8, SHMT1 and β-ACTIN by immunoblotting (right). (**d**) PEO4 cells expressing NS or *SHMT1* shRNAs alone or expressing *IL-6* or *IL-8* cDNA were analyzed for anchorage-independent growth in a soft-agar assay. Representative images under indicated conditions (top) and colony number and size relative to those of cells expressing NS shRNA (bottom) are shown. (**e**) PEO4 cells expressing NS or *SHMT1* shRNAs alone or expressing *IL-6* or *IL-8* cDNA were injected subcutaneously into the flanks of athymic nude mice. Average tumor volumes (*n*=5) at indicated times are shown. Data are presented as mean±s.e.m.; *P<0.05 and **P<0.005.

To determine the effect of *SHMT1* knockdown on expression of mRNAs of protein-coding genes in ovarian cancer cells, we performed global mRNA expression analysis in PEO4 ovarian cancer cells after *SHMT1* knockdown. *SHMT1* knockdown resulted in downregulation of 46 genes and upregulation of 9 genes (Supplementary Table 2). Ingenuity biological pathway analysis identified IL-6 and IL-8 (also known as CXCL8) as belonging to the biological pathways most significantly altered by *SHMT1* knockdown (Supplementary Table 3). IL-6 and IL-8 are inflammatory cytokines that exert tumor-promoting activity.^22, 23, 24^ We confirmed the results of our gene expression array analysis and noted that, similar to those results, SHMT1 loss resulted in significant downregulation of *IL-6* and *IL-8* mRNA in PEO4 cells (Figure 5a).

To determine if *SHMT1* loss-mediated downregulation of IL-6 and IL-8 also occurs in other ovarian cancer cell lines, we measured *IL-6* and *IL-8* mRNA levels in other ovarian cancer cell lines COV504 and COV413B expressing *SHMT1* shRNA and observed similar results (Figures 5b and c). Finally, to determine the role of IL-6 and IL-8 in mediating the tumor-promoting effect of *SHMT1*, we performed rescue experiments. To this end, we asked if ectopic expression of IL-6 or IL-8 restores tumor growth in ovarian cancer cells expressing *SHMT1* shRNA. Our results showed that expression of either IL-6 or IL-8 partially rescued growth inhibition caused by *SHMT1* knockdown in cell culture (Figure 5d) and in mice (Figure 5e). Collectively, these findings demonstrate that loss of SHMT1 results in decreased expression of the pro-oncogenic inflammatory cytokines IL-6 and IL-8, which in turn results in inhibition of ovarian cancer tumor growth.

### Sialic acid Neu5Ac stimulates IL-6 and IL-8 expression and promotes ovarian cancer tumor growth and cell migration

Our results showed that ectopic expression of IL-6 or IL-8 was, in part, able to rescue the phenotypes resulting from the loss of SHMT1. Additionally, our metabolomic analysis showed that SHMT1 loss results in reduced expression of metabolites of the nucleotide sugar and amino sugar metabolic pathway, including sialic acid Neu5Ac. Previous studies indicated an important role of sialic acid in cancer initiation and progression.^20, 21^ Interestingly, several oncogenes and other cancer-related genes are subject to sialylation, which modulates their biological functions and activities.^21^

Furthermore, a recent study demonstrated correlation of multiple different oncogene-mediated transformation events with changes in the amino sugar and nucleotide sugar metabolic pathway.^25^ This study not only identified changes in amino sugar and nucleotide sugar metabolism but also showed that the gene signature of cytidine monophosphate *N*-acetylneuraminic acid synthetase knockdown overlapped significantly with that of our *SHMT1* knockdown gene expression array (Supplementary Table 4).^25^ *N*-acetylneuraminic acid synthetase encodes *N*-acetylneuraminate cytidylyltransferase that catalyzes the conversion of Neu5Ac to CMP-Neu5Ac (also known as CMP-sialic acid), which in turn participates in protein sialylation. Similar to our results with ovarian cancer cells expressing *SHMT1* shRNA, loss of *N*-acetylneuraminic acid synthetase resulted in reduced levels of IL-6 and IL-8 (Supplementary Table 4).

**Figure 6:**
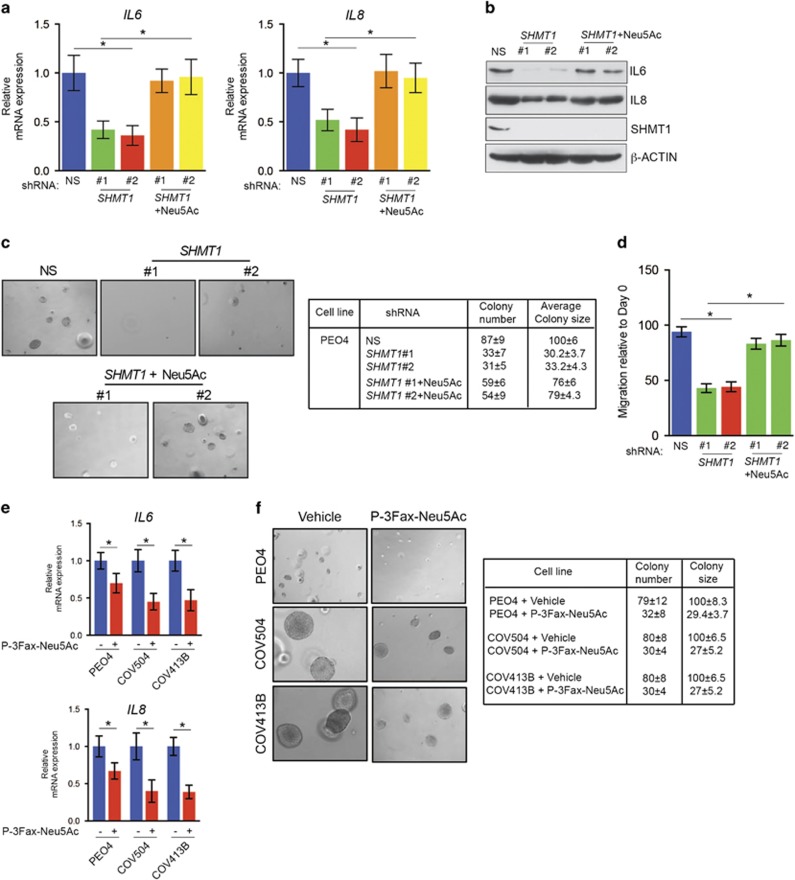
Neu5Ac supplementation stimulates IL-6 and IL-8 and rescues growth of SHMT1 knockdown ovarian cancer cells (**a**) and (**b**) PEO4 cells expressing indicated shRNAs without or with Neu5Ac were analyzed for expression of *IL-6* and *IL-8* mRNA by RT–qPCR (**a**) and of *IL-6*, *IL-8*, SHMT1 and β-ACTIN by immunoblotting (**b**). (**c**) PEO4 cells expressing indicated shRNAs without or with Neu5Ac were tested for the ability to grow in an anchorage-independent manner in a soft-agar assay. Representative images (top) and colony number and size relative to those of cells expressing nonspecific (NS) shRNA (bottom) are shown. (**d**) PEO4 cells expressing indicated shRNAs without or with Neu5Ac were tested for their ability to migrate in wound-healing assay. Migration on day 4 relative to that on day 0 under indicated conditions is shown. (**e**) Indicated ovarian cancer cells were treated with vehicle (1 × PBS) or Neu5Ac-targeting glycomimetic P-3Fax-Neu5Ac and analyzed for expression of *IL-6* and *IL-8* mRNA by RT–qPCR. Expression relative to that in control vehicle-treated cells is shown (**f**). Indicated ovarian cancer cells were treated with vehicle or P-3Fax-Neu5Ac and analyzed for ability to grow in an anchorage-independent manner in a soft-agar assay. Representative soft-agar assay images for indicated cell lines treated with vehicle or P-3Fax-Neu5Ac are shown (left) and relative colony number and average colony size are shown (right). Data are presented as mean±s.e.m.; *P<0.05.

Based on this evidence, we investigated whether Neu5Ac supplementation could rescue loss-of-*SHMT1*-mediated tumor cell growth inhibition and reduced migration phenotypes. To this end, we measured levels of *IL-6* and *IL-8* mRNA and protein after Neu5Ac supplementation of PEO4 cells expressing *SHMT1* shRNAs. Addition of Neu5Ac resulted in increased levels of *IL-6* and *IL-8* mRNA (Figure 6a) and protein (Figure 6b) in ovarian cancer cells expressing *SHMT1* shRNA.

We next performed rescue experiments and tested whether addition of Neu5Ac could rescue the ability of PEO4 cells expressing *SHMT1* shRNA to grow in soft agar and enhance their migratory ability. Supplementation with Neu5Ac rescued the ability of PEO4 cells expressing *SHMT1* shRNA to form colonies in a soft-agar assay (Figure 6c). In addition, we were able to rescue the migration phenotype of PEO4 cells expressing *SHMT1* shRNA (Figure 6d).

A previous study showed that Neu5Ac can be targeted using a glycomimetic P-3Fax-Neu5Ac to inhibit metastatic spread of melanoma.^26^ Because our results showed that Neu5Ac was necessary to facilitate ovarian cancer growth, we investigated the ability of the previously described Neu5Ac-blocking glycomimetic P-3Fax-Neu5Ac to block IL-6 and IL-8 expression and inhibit ovarian cancer tumor cell growth. Treatment with P-3Fax-Neu5Ac significantly inhibited *IL-6* and *IL-8* mRNA expression (Figure 6e) and the ability of ovarian cancer cells to grow in an anchorage-independent manner in a soft-agar assay (Figure 6f). Collectively, these results demonstrate that Neu5Ac induces the inflammatory cytokines IL-6 and IL-8, which in turn promote ovarian cancer tumor growth. These studies also identified SHMT1-mediated regulation of Neu5Ac as a genetic vulnerability of ovarian cancer cells that can be targeted by approaches aimed at inhibition of Neu5Ac and by potentially inhibiting SHMT1 using specific inhibitors.

## Discussion

**Figure 7:**
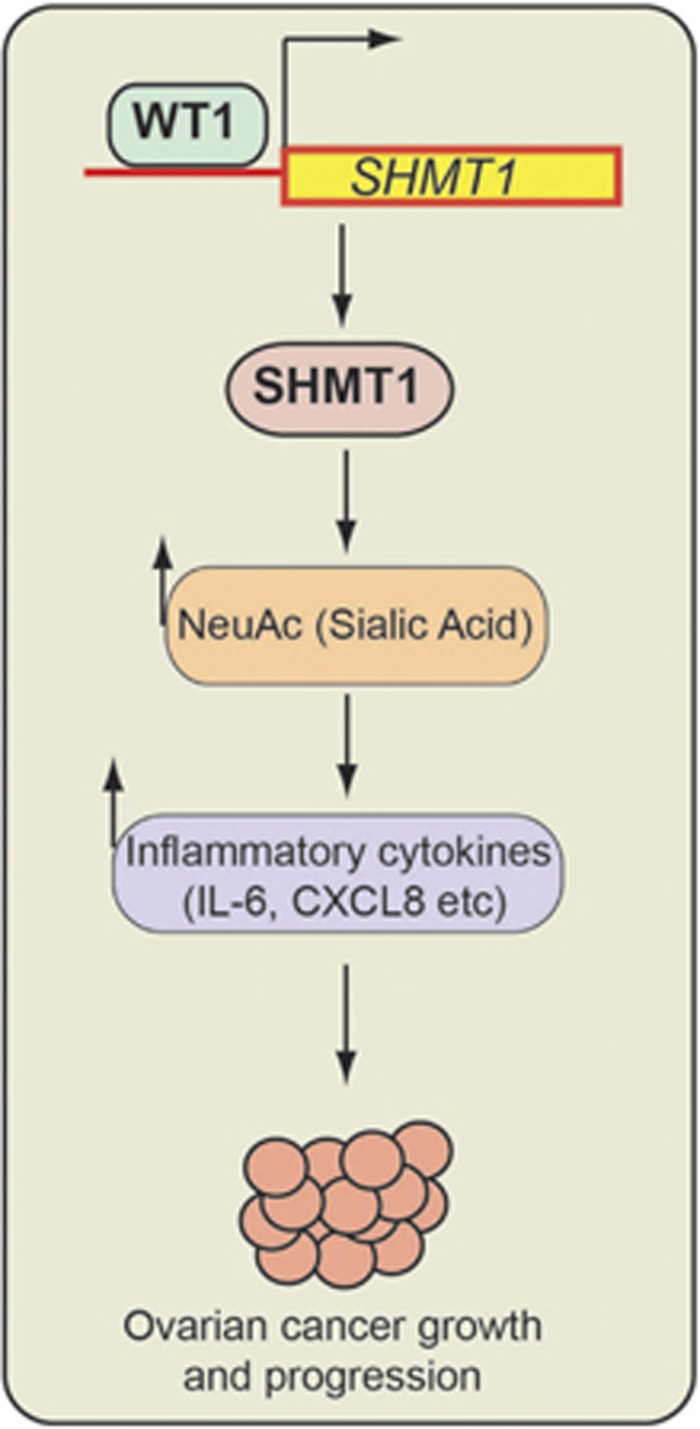
Model. SHMT1, by regulating nucleotide and amino sugar metabolism, regulates pro-oncogenic inflammatory cytokine expression through the sialic acid Neu5Ac, which in turn facilitates ovarian cancer cell growth and migration.

Our results allow us to draw several important conclusions that are summarized in Figure 7 and described below. First, SHMT1 is necessary for ovarian cancer tumor growth and progression, and SHMT1 expression is regulated by transcription factor WT1. Second, in addition to the expected alterations in the metabolites of the folic acid metabolic pathway, *SHMT1* knockdown in ovarian cancer cells unexpectedly resulted in downregulation of several metabolites of the amino sugar and nucleotide sugar metabolic pathway, including Neu5Ac. Third, SHMT1 promotes ovarian cancer tumor growth and progression, in part, by facilitating expression of pro-oncogenic inflammatory cytokines IL-6 and IL-8, and, at least in part, by regulating cellular levels of Neu5Ac. These findings identify SHMT1 and Neu5Ac as important precision therapy targets for treatment of ovarian cancer.

Although we found that six genes of the folic acid metabolic pathway were overexpressed in the ovarian cancer samples compared with normal ovary, no other gene knockdown was as effective in inhibiting the growth of ovarian cancer cells as was the knockdown of *SHMT1*. This finding underpins the importance of functional validation experiments such as ours to distinguish passenger transcriptional changes from driver transcriptional changes in cancer cells. Overall, our experimental approach also provides a general framework for performing similar experiments to identify important metabolic drivers of tumor growth.

### SHMT1 as a new regulator of ovarian cancer tumor growth and cell migration

Metabolic enzymes and pathways have emerged as important enablers of cellular acquisition of the hallmarks of cancer.^4^ Two previous studies have shown that SHMT1 is necessary for lung tumor growth.^27, 28^ Additionally, polymorphisms in the *SHMT1* gene have been shown to be associated with a wide variety of cancers, including childhood acute lymphoblastic leukemia, non-Hodgkins lymphoma, head and neck cancers, and colon cancer.^29, 30, 31, 32^ In this study, we identified SHMT1 as a new regulator of ovarian cancer tumor growth and progression.

### SHMT1 connects metabolic alterations with regulation of pro-oncogenic inflammatory cytokines

*SHMT1* encodes the cytoplasmic form of SHMT, a pyridoxal phosphate-containing enzyme that catalyzes the reversible conversion of serine and tetrahydrofolate to glycine and 5, 10 methylene tetrahydrofolate.^33^ This in turn provides one-carbon units for the production of methionine, thymidylate and purines in the cytoplasm. Our metabolomic analysis identified multiple alterations in metabolic pathways, including in amino sugar and nucleotide sugar metabolism intermediates, after *SHMT1* knockdown. In particular, we found that *SHMT1* knockdown resulted in reduced levels of Neu5Ac. Additionally, transcriptome-wide mRNA expression analysis of ovarian cancer cells lacking SHMT1 revealed reduced *IL-6* and *IL-8* mRNA levels. We found that Neu5Ac supplementation of culture medium stimulated IL-6 and IL-8 production and rescued the ability of ovarian cancer cells expressing *SHMT1* shRNA to grow in an anchorage-independent manner and increased their migratory ability. Collectively, these results demonstrate that reduced levels of Neu5Ac after SHMT1 downregulation attenuate production of pro-oncogenic inflammatory cytokines, which consequently blocks ovarian cancer tumor growth and progression.

It is possible that SHMT1 might regulate other cellular factors beyond what we described here that may contribute to its ability to promote ovarian cancer tumor growth and migration. In this direction, we noticed some interesting candidates in our gene expression profiling data of ovarian cancer cells expressing *SHMT1* shRNAs. In particular brain-derived neurotrophic factor (BDNF) and transcription factor activator protein-2α might be of interest for additional studies as potential mediators of ovarian cancer-promoting function of SHMT1. We find that knockdown of *SHMT1* results in reduced *BDNF* expression and increase in *transcription factor activator protein-2α* expression in ovarian cancer cells. BDNF has been show to promote the growth of many different types of cancers.^34, 35, 36^ In particular, BDNF has been shown to prolong Tropomyosin receptor kinase B activation, and thereby promotes non-small-cell lung cancer growth.^34^ Contrary to BDNF, transcription factor activator protein-2α has been shown to exert tumor-suppressive effects in cancer cells.^37, 38, 39^ Therefore, it is possible that of the ability of SHMT1 to promote ovarian cancer tumor growth and migration might stem from its ability to stimulate BDNF expression and repress transcription factor activator protein-2α expression. Future studies in this direction will likely provide a more definitive answer.

## Materials and methods

### Cell culture, shRNAs, transfection, and retrovirus and lentivirus preparation

European Collection of Authenticated Cell Culture HGS ovarian cancer cell lines PEO4, COV504 and COV413B were obtained from Sigma-Aldrich (St Louis, MO, USA) and cultured according to European Collection of Authenticated Cell Culture recommendations. All the cell lines were mycoplasma free and were authenticated by STR profiling. *SHMT1*, *IL-6* and *IL-8* cDNAs were purchased from Origene (Rockville, MD, USA).

Lentiviral shRNA plasmids were obtained from Open Biosystems. shRNA ID information is provided in Supplementary Table 5. For retrovirus or lentivirus production, constructs and viral packaging plasmids were co-transfected into 293T cells using Effectene (Qiagen, Hilden, Germany) following the supplier’s recommendations. Viral supernatants were collected 48 h after transfection, and purified virus particles were used to infect ovarian cancer cell lines. Cells were selected on puromycin to enrich for cells expressing shRNAs.

### Analysis of TCGA HGS ovarian cancer mRNA expression data

To identify metabolic genes that are significantly upregulated in HGS ovarian cancer samples compared with normal ovary, we analyzed the TCGA ovarian cancer data set using Oncomine (Thermo Fisher Scientific, Waltham, MA, USA). We identified the top 10% of a total of 1262 significantly overexpressed genes in the data set and compared them with genes implicated in regulation of cellular metabolism using a comprehensive list of 2752 genes encoding human metabolic enzymes and transporters.^40^ In total, we identified 157 genes regulating metabolic pathways that were in the top 10% of significantly overexpressed genes. Ingenuity biological pathway analysis revealed that several of these genes were significantly associated with three major metabolic pathways, that is, oxidative phosphorylation, histidine-glutamate-glutamine metabolic pathway and the folic acid metabolism pathway.

### Microarray experiments and data analysis

For microarray experiments using PEO4 cells, total RNA was isolated from PEO4 cells expressing either a control nonspecific shRNA or one of two *SHMT1* shRNA sequences and used to generate labeled antisense RNA. All antisense RNAs were made using the Ambion MessageAmp Kit (Thermo Fisher Scientific) and hybridized to the Illumina HumanHT-12 V4.0 (Illumina, San Diego, CA, USA) expression BeadChip using Illumina’s protocol.

The microarray data were processed using GenomeStudio (Illumina), log 2-transformed and quantile-normalized using the lumi package of Bioconductor. All samples passed quality-control assessment, which included checking various control plots as suggested by Illumina, as well as other standard microarray-related analyses. Differential expression analyses were performed using the limma package, and a moderated *t*-test with a Benjamini–Hochberg multiple testing correction procedure was used to determine statistical significance (adjusted *P*-value <0.05). Pathway analysis of differentially expressed genes for each comparison was performed using MetaCore (version 6.8 build 29806; GeneGo, New York, NY, USA). Microarray data were submitted to Gene Expression Omnibus (accession number: GSE76440).

### Metabolomic analysis

PEO4 cells expressing *SHMT1* or control nonspecific shRNA were analyzed for metabolic pathway alterations using the capillary electrophoresis time-of-flight mass spectrometry-based basic scan profiling method of Human Metabolome Technologies (Boston, MA, USA). Cells (1 × 10^6^) for each condition in duplicate were analyzed by this method, and samples were prepared as per the recommendations of Human Metabolome Technologies (Cambridge, MA, USA). For data analysis, peaks detected in capillary electrophoresis time-of-flight mass spectrometry analysis were extracted using automated integration software (MasterHands version 2.16.0.15 developed at Keio University, Tokyo, Japan) to obtain mass/charge ratio (*m*/*z*), migration time and peak area. Peak area was then converted to relative peak area using the following equation: relative peak area=metabolite peak area/internal standard peak area × number of cells. The peak detection limit was determined based on signal-to-noise ratio=3. Putative metabolites were then assigned from the Human Metabolomic Technologies standard library and known–unknown peak library on the basis of *m*/*z* and migration time. All metabolite concentrations were calculated by normalizing the peak area of each metabolite with respect to the area of the internal standard and by using standard curves, which were obtained by single-point (100 μM) calibrations. The profile of peaks of putative metabolites was represented on metabolic pathway maps using Visualization and Analysis of Networks containing Experimental Data (VANTED) software (http://vanted.ipk-gatersleben.de/).

### RNA preparation, cDNA synthesis and RT–qPCR analysis

For mRNA expression analyses, total RNA was extracted with TRIzol (Invitrogen, Carlsbad, CA, USA) and purified using RNeasy mini columns (Qiagen). cDNA was generated using the M-MuLV First-Strand cDNA Synthesis Kit (New England Biolabs, Ipswich, MA, USA) according to the manufacturer’s instructions. Quantitative reverse transcription–PCR (RT–qPCR) was performed using the Power SYBR Green Master Mix (Applied Biosystems, Foster City, CA, USA) according to the manufacturer’s instructions. Actin was used as an internal control. Primer sequences are provided in Supplementary Table 5.

### Chromatin immunoprecipitation

The *SHMT1* promoter sequence was downloaded from the UCSC genome browser and analyzed using rVisa 2.0 (REF). Chromatin immunoprecipitation experiments were performed as described previously.^41^ Normalized Ct (ΔCt) values were calculated by subtracting the Ct obtained with input DNA from that obtained with immunoprecipitated DNA (ΔCt=Ct(IP)−Ct(input)). Relative fold enrichment of a factor at the target site was then calculated using the formula 2^−(ΔCt(T)^^−ΔCt(Actb))^, where ΔCt(T) and ΔCt(Actb) are ΔCt values obtained using target and β-*ACTIN* (negative control) primers, respectively.

### Antibodies and immunoblot analysis

Immunoblot analysis was performed as described previously.^42^ Blots were developed using the Thermo Scientific SuperSignal West Pico Chemiluminescent Substrate or SuperSignal West Femto Maximum Sensitivity Substrate (Thermo Scientific, Waltham, MA, USA), as appropriate. The details of antibodies are provided in Supplementary Table 5.

### Soft-agar and mouse tumorigenesis assays

For the soft-agar assay, individual cell lines were seeded in triplicate at three different dilutions, ranging from 5 × 10^3^ to 2 × 10^4^ cells in 6-well plates. Cells were seeded into a layer of 0.4% soft agar. After 4–5 weeks of culture, images of colonies that had formed were captured using an inverted light microscope. Colonies were stained with 0.005% crystal violet and counted, and the average area of each replicate was calculated using the ImageJ software (ImageJ, National Institutes of Health, Bethesda, MD, USA) and plotted. Each experiment was repeated at least two times. Athymic nude (NCr nu/nu) female mice (6 weeks of age) were injected subcutaneously with cells expressing various shRNA or shRNA and cDNA combinations as described in the text and shown in figures. Mouse number per group were determined based on power analysis. Tumor volume was measured every week and was calculated using the formula: length × width^2^ × 0.5. All animal protocols were approved by the Institutional Animal Care and Use Committee (IACUC) at Yale University.

### Wound-healing assay

For the wound-healing assay, 5 × 10^5^ ovarian cancer cells were seeded in each well of a 12-well plates. Cells were cultured until they reached 85% confluence at 37 °C in a humidified atmosphere of 5% CO_2_. A wound was made by scratching the monolayer with a sterile micropipette tip. Plates were then washed two times with 37 °C phosphate-buffered saline (PBS) for ~30 s each to remove floating cells, followed by the addition of fresh culture medium. The region of the wound was marked on the outer surface of the bottom of the plate, and four fields were photographed for each wound and plate at time 0 using an inverted light microscope. The same wound fields were photographed again on day 4.

### Invasion assay

The invasion assay was carried out in a BioCoat Growth Factor Reduced Matrigel Invasion Chamber (BD Biosciences, San Jose, CA, USA; cat. no. 354483) using ovarian cancer cells carrying nonspecific or *SHMT1* shRNAs. Briefly 5 × 10^4^ cells/insert were seeded in triplicate in low-serum medium after 6 h of serum starvation. The cells were allowed to migrate towards serum-rich medium in the bottom well for 20 h. The number of cells migrating through the Matrigel was quantified by imaging after DAPI (4',6-diamidino-2-phenylindole) staining; 8–12 fields per membrane were counted, and quantification of nuclei was performed using the ImageJ software (NIH).

### Treatment of cells with Neu5Ac and sialic acid-blocking glycomimetic P-3Fax-Neu5Ac

To measure IL-6 and IL-8 levels after Neu5Ac treatment, 3 × 10^5^ ovarian cancer cells were seeded in each well of a 6-well plate. After reaching 85% confluence, the cells were washed with PBS for 30 min followed by replacement of PBS with serum-free Opti-MEM containing either Neu5Ac (10 mM) or vehicle (1 × PBS) and incubated for 2 h. Cells were harvested and total RNA was prepared using TRIzol (Invitrogen) as recommended by the supplier and used for RT–qPCR as described above. To measure IL-6 and IL-8 after P-3Fax-Neu5Ac treatment, 2 × 10^5^ ovarian cancer cells were seeded in each well of a 6-well plate and cultured for 24 h in complete medium. Cells were then treated for 3 days with Opti-MEM containing 23.1 μg/ml P-3Fax-Neu5Ac or vehicle control. After the treatment cells were harvested, and total RNA was prepared using TRIzol (Invitrogen) as recommended by the supplier and used for RT–qPCR as described above. For soft-agar assay with Neu5Ac supplementation, cells were treated with Neu5Ac (10 mM) two times weekly for 4 weeks. For soft-agar assay with P-3Fax-Neu5Ac treatment, cells were treated with 23.1 μg/ml P-3Fax-Neu5Ac two times weekly for about 4 weeks. Soft-agar assay and analysis was performed as described above.

### Statistical analysis

All experiments used three biological replicates. Results of individual experiments are expressed as mean±s.e.m. The significance between the mean values for each study was evaluated by two-tailed unpaired Student’s *t*-test. GraphPad Prism version 6.0 h for Macintosh (http://www.graphpad.com) was used for all the analyses.
