# Supplementary material for: Serine hydroxymethyl transferase 1 stimulates pro-oncogenic cytokine expression through sialic acid to promote ovarian cancer tumor growth and progression
Source: Oncogene. 2017 Mar 13;36(28):4014–24. doi: 10.1038/onc.2017.37 (PMC5509519; doi:10.1038/onc.2017.37)
Supplement: Supplementary Material [file onc201737x1.pdf]

**Supplementary Figure 1**

**A**

Significantly altered metabolic pathways

| Pathway maps | p-values | Ratios (altereted gene/total genes) |
|---|---|---|
| Oxidative phosphorylation | 1.210E-6 | 10/105 |
| Folic acid metabolism | 6.858E-5 | 6/53 |
| Histidine-glutamate -glutamine metabolism | 2.648E-4 | 7/95 |

**B**

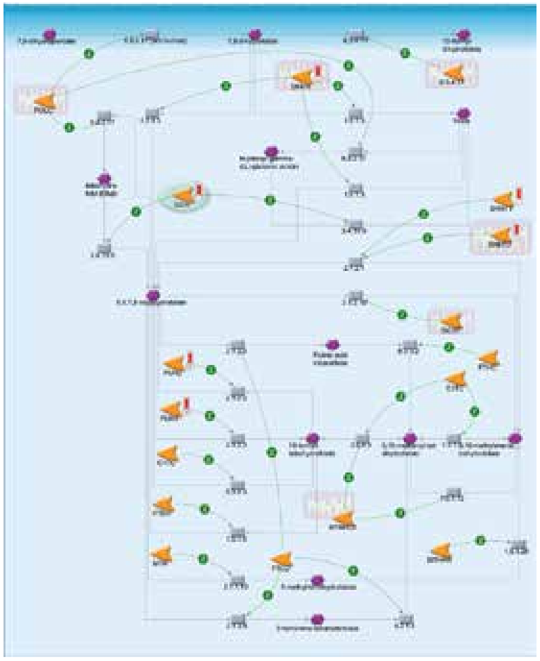

**C**

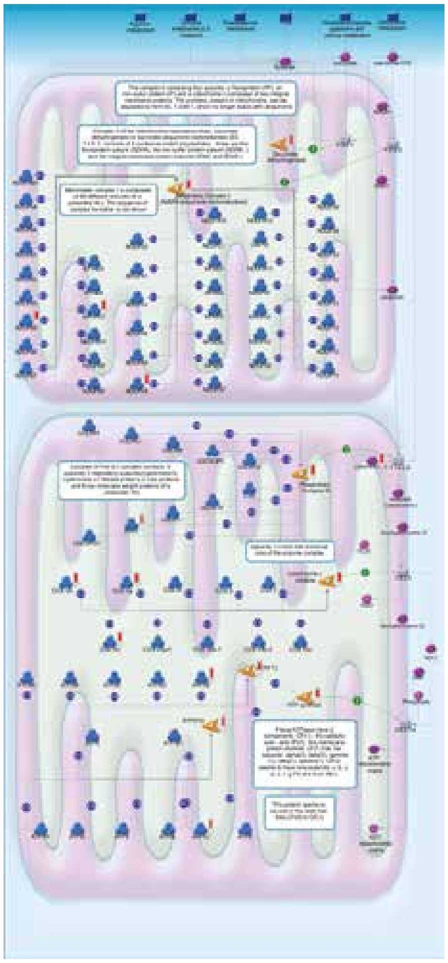

**D**

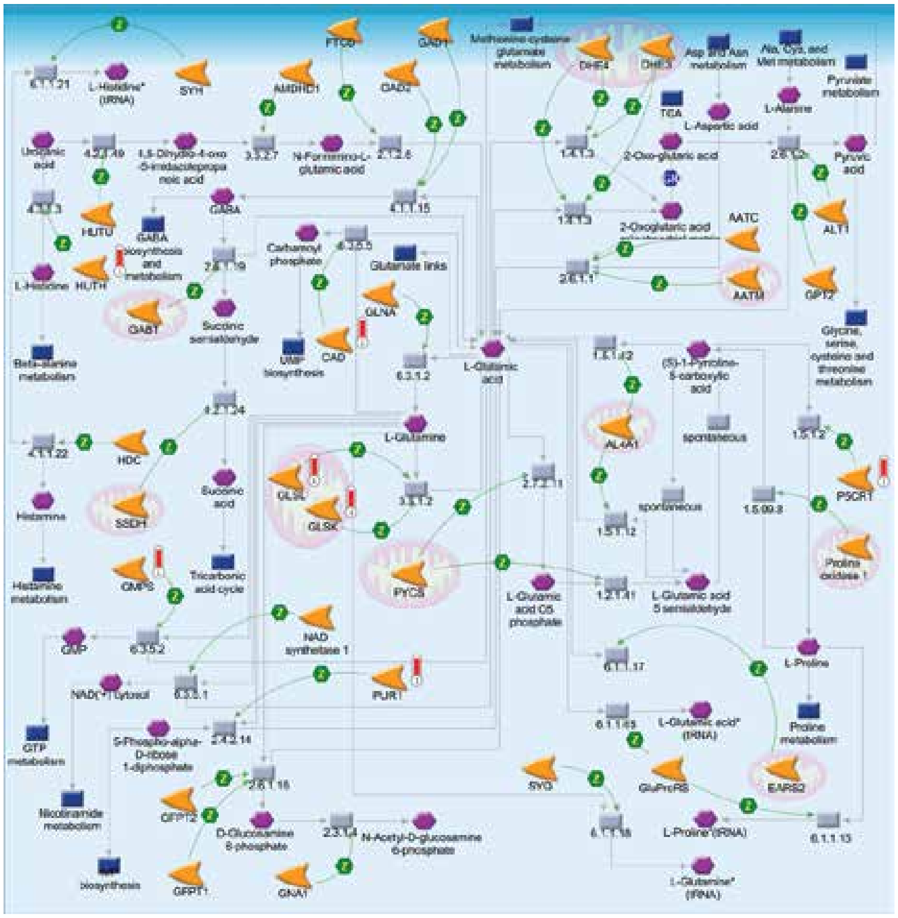

# Supplementary Figure 2

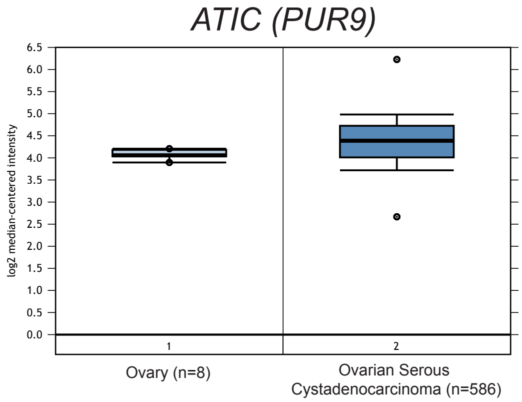


*ATIC* Fold change = +1.227
p-value = 8.49E-6

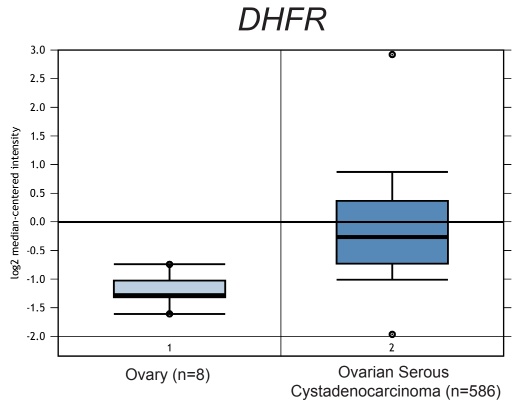


*DHFR* Fold change = +2.084
p-value = 1.27E-6

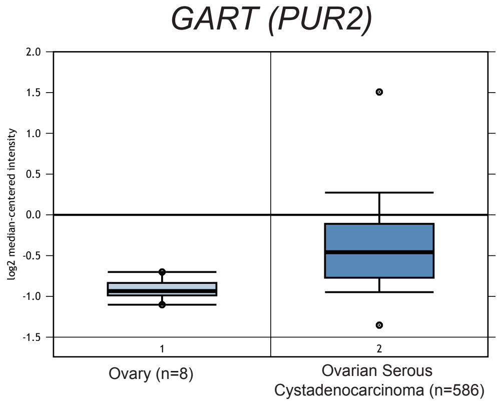


*GART* Fold change = +1.446
p-value = 4.73E-7

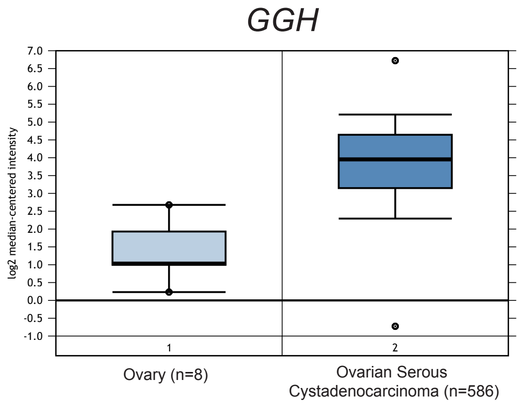


*GGH* Fold change = +6.21
p-value = 1.03E-5

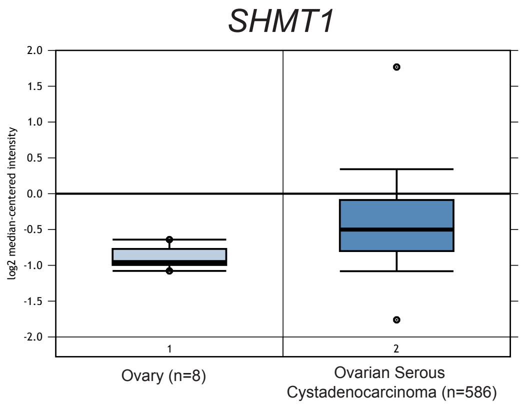


*SHMT1* Fold change = +1.41
p-value = 2.26E-6

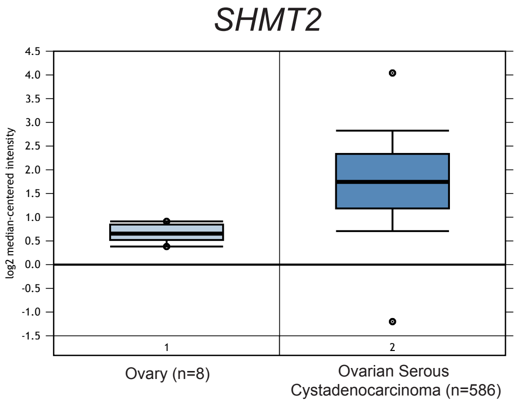


*SHMT2* Fold change = +2.163
p-value = 1.13E-8

**A**

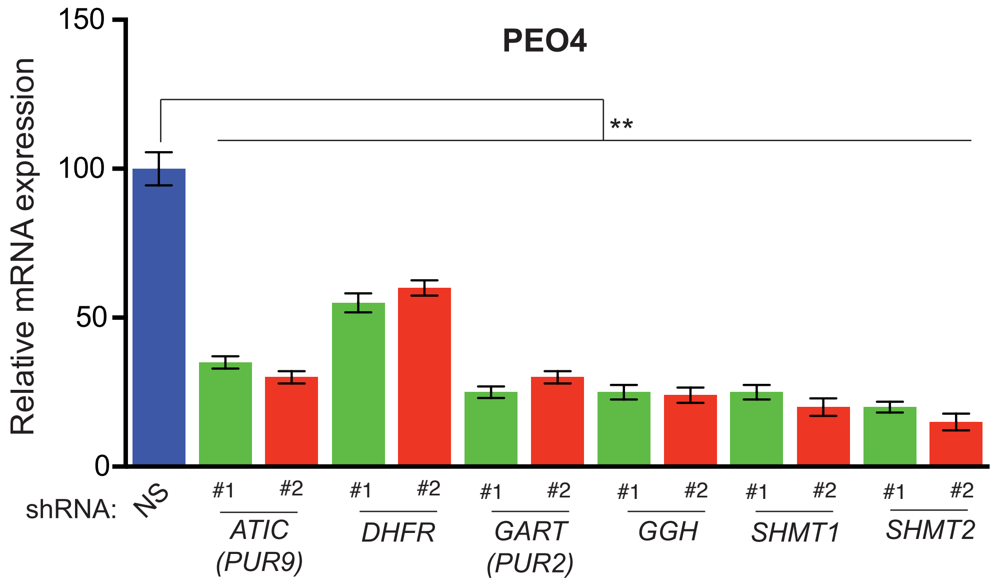
PEO4
Relative mRNA expression
150
100
50
0
**
shRNA:
NS
#1
#2
ATIC (PUR9)
DHFR
GART (PUR2)
GGH
SHMT1
SHMT2

**B**

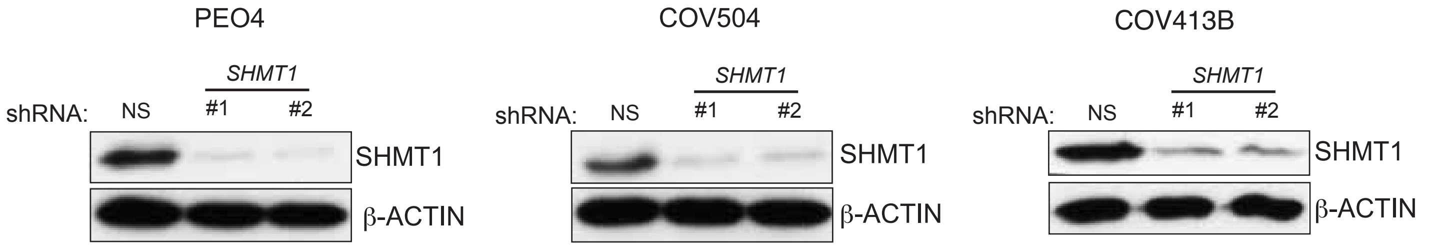
PEO4
COV504
COV413B
shRNA:
NS
SHMT1
#1
#2
SHMT1
β-ACTIN

## Supplementary Figure 4

**A**

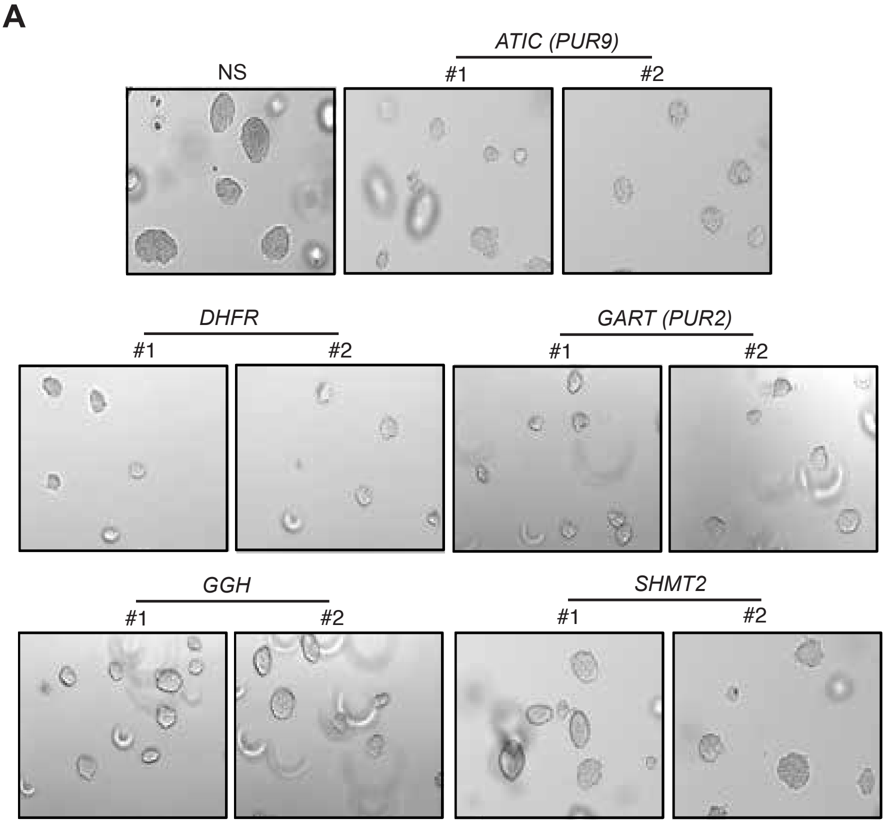


**B**

| Cell line | shRNA | Colony number | Average Colony size |
|---|---|---|---|
| PEO4 | NS | 93±8.2 | 100±4.8 |
| | *ATIC (PUR9)*#1 | 60±6 | 34.2±3.2 |
| | *ATIC (PUR9)*#2 | 50±7.3 | 38.4±3 |
| | *DHFR*#1 | 50±6.3 | 34.6±4.1 |
| | *DHFR*#2 | 70±7.2 | 35.5±3.4 |
| | *GART (PUR2)*#1 | 70±5.6 | 30.7±3.5 |
| | *GART (PUR2)*#2 | 60±6.6 | 33.8±4.2 |
| | *GGH*#1 | 90±11 | 53.8±3.8 |
| | *GGH*#2 | 70±10 | 65.3±4.8 |
| | *SHMT2*#1 | 80±9 | 92.3±4 |
| | *SHMT2*#2 | 60±8 | 96.1±4.3 |

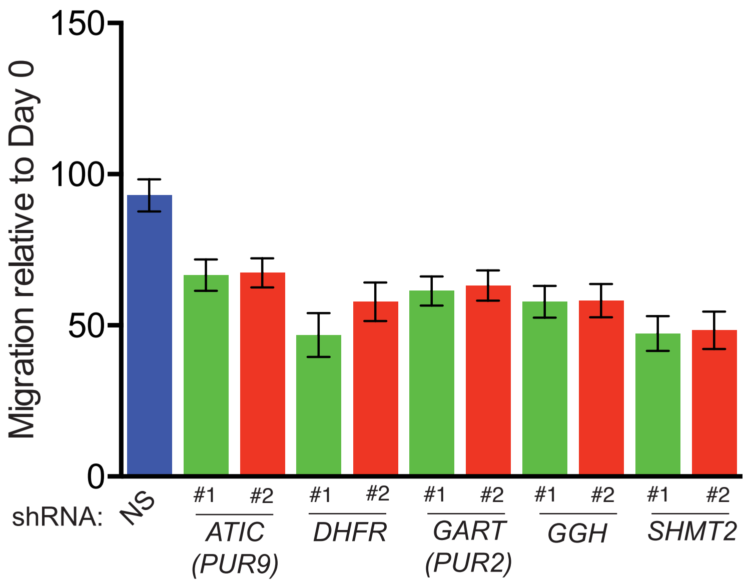
150
100
50
0
Migration relative to Day 0
shRNA:
NS
#1
#2
ATIC
(PUR9)
DHFR
GART
(PUR2)
GGH
SHMT2

**Supplementary Figure 6**

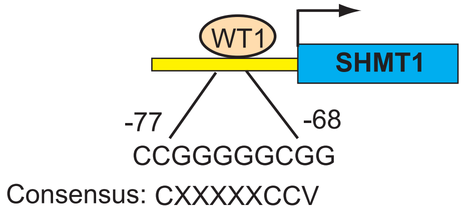

**Supplementary Figure Legends**

**Supplementary Figure 1. Identification of key metabolic pathway alterations in ovarian cancer. A.** Ingenuity pathway analysis (IPA) identifies oxidative phosphorylation, folic acid metabolism, and histidine-glutamate-glutamine metabolism as three key metabolic pathways that were altered in ovarian cancer cells. The table lists p-values and the number of genes in each pathway in ratio with genes that are altered in indicated metabolic pathways. **B.** Folic acid metabolic pathway showing six genes marked with red stick image (**inset**) that are significantly upregulated in TCGA ovarian cancer datasets. **C.** Oxidative phosphorylation pathway showing 10 genes marked with red stick image (**inset**) that are significantly upregulated in TCGA ovarian cancer datasets. **D.** Histidine-glutamate-glutamine metabolic pathway showing seven genes marked with red stick image (**inset image**) that are significantly upregulated in TCGA ovarian cancer datasets.

**Supplementary Figure 2. Genes in folic acid metabolic pathway are upregulated in ovarian cancer cells.** TCGA ovarian cancer dataset analysis shows relative fold-upregulation and p-values for indicated genes.

**Supplementary Figure 3. Knockdown of genes of folic acid metabolic pathway in multiple ovarian cancer cell lines. A.** PEO4 cells expressing either control nonspecific (NS) shRNA or shRNA targeting the indicated mRNAs were analyzed for expression of indicated mRNAs by RT-qPCR. Expression level relative to that of cells expressing NS shRNA is shown. **B.** PEO4,

COV504, and COV413B cells expressing NS or *SHMT1* shRNAs were analyzed for SHMT1 and β-ACTIN expression by immunoblotting. Data are presented as mean ± SEM; **$p<0.005$.

**Supplementary Figure 4. Effect of knockdown of folic acid metabolic pathway genes on ovarian cancer growth.** PEO4 cells expressing either control nonspecific (NS) shRNA or shRNA targeting the indicated mRNAs were analyzed for the ability to grow in an anchorage-independent manner in a soft-agar assay. Representative soft-agar assay images (**A**) and colony number and size relative to those of cells expressing NS shRNA (**B**) are shown.

**Supplementary Figure 5. Effect of knockdown of folic acid metabolic pathway genes on ovarian cancer cell migration.** PEO4 expressing either a control nonspecific (NS) or indicated targeted shRNAs were analyzed for the ability to migrate in a wound-healing assay. Representative cell migration for indicated shRNAs at day 4 relative to day 0 is shown.

**Supplementary Figure 6. Schematic representation of transcription factor Wilms tumor 1 (WT1) binding site on *SHMT1* promoter.**

**Supplementary Table 1. List of 6 genes regulating folic acid metabolic shortlisted for functional validation in ovarian cancer samples**

| Gene Symbol | Gene Name | Functional Category | Fold upregulation in TCGA dataset |
|---|---|---|---|
| ATIC (PUR9) | 5-aminoimidazole-4-carboxamide ribonucleotide formyltransferease/IMP cyclohydrolase | Folic Acid Metabolism | **+1.227** p-value=8.49E-6 |
| DHFR | Dihydrofolate reductase | Folic Acid Metabolism | **+2.084** p-value=1.27E-6 |
| GART (PUR2) | phosphorbiosylglycinamide formyltransferase, phosphorbiosylglycinamide synthetase, phosphorbiosylaminoimidazole synthetase | Folic Acid Metabolism | **+1.446** p-value=4.73E-7 |
| GGH | Gamma-glutamyl hydrolase | Folic Acid Metabolism | **+6.21** p-value=1.03E-5 |
| SHMT1 | Serine Hydroxymethyltransferase 1 | Folic Acid Metabolism | **+1.41** p-value=2.26E-6 |
| SHMT2 | Serine Hydroxymethyltransferase 2 | Folic Acid Metabolism | **+2.163** p-value= 1.13E-8 |

**Supplementary Table 2. Fold change for significantly altered genes in PEO4 cells expressing *SHMT1* shRNAs compared to the cells expressing non-silencing shRNA**

| Gene Symbol | Fold change *SHMT1* shRNA#1 | Fold Change *SHMT1* shRNA#2 |
|---|---|---|
| ADM | -1.51119176 | -1.36910868 |
| ATOH8 | 1.32527115 | 1.715921298 |
| AVPI1 | -1.282157373 | -1.512357676 |
| BDNF | -1.606796987 | -1.338330091 |
| BLACAT1 | -1.334442404 | -1.331317824 |
| C3orf14 | -1.483782624 | -1.67738232 |
| CAMK2N1 | -1.932333413 | -1.825005487 |
| CD163L1 | -1.400289792 | -1.574268376 |
| CD24 | -1.531860014 | -1.997061865 |
| CD24 | -1.531860014 | -1.492119753 |
| CFI | -1.518543288 | -1.365039684 |
| CXCL8 | -1.342219044 | -2.201465452 |
| CXCL8 | -1.342219044 | -2.164724636 |
| DCAKD | 1.475621422 | 1.294620178 |
| DEFB1 | -1.730575 | -1.77585153 |
| DHRS9 | -1.451458765 | -1.563242937 |
| FAM83D | 1.382984166 | 1.260764423 |
| FGFR3 | 1.466252262 | 1.372789166 |
| FMOD | 1.346585943 | 1.357794286 |
| GFRA2 | 3.237919393 | 2.026628216 |
| ICAM2 | -1.338111374 | -1.379860057 |
| IER3 | -1.903510432 | -1.899100239 |
| IL24 | -1.485749921 | -1.703134679 |
| IL6 | -1.528866418 | -1.316266015 |
| KANK4 | -1.51939388 | -1.554746992 |
| KANK4 | -1.51939388 | -1.545027157 |
| KRT17 | -1.385708821 | -1.25350592 |
| KRT81 | -1.811243849 | -1.865098126 |
| LEMD1 | -1.576916891 | -1.398655882 |
| MAL | -2.606955795 | -1.441467059 |
| MGAT3 | -1.255031755 | -1.546480376 |
| MT1M | -1.786099952 | -1.507042657 |
| NDN | -1.273268363 | -1.442652115 |
| NT5E | -1.491530971 | -1.560153367 |
| PBX3 | -1.370465743 | -1.521449612 |
| PDE4B | -1.387581731 | -2.273554584 |
| PDE4B | -1.387581731 | -1.438673871 |
| PHLDA1 | -1.391449138 | -1.608509464 |
| PTGES | -1.600225599 | -1.322914701 |
| PXDN | -1.311852644 | -1.604810459 |
| RARRES1 | -1.862862733 | -2.006348736 |
| RARRES1 | -1.862862733 | -1.697142428 |
| RASD1 | 1.724253894 | 1.360291829 |
| S100P | -1.936451328 | -1.878168746 |
| SHMT1 | -1.726555704 | -1.238954893 |
| SHMT1 | -1.223038773 | -1.025780892 |

| | | |
|---|---|---|
| SLC2A3 | -2.620649702 | -1.389633758 |
| SRPX2 | -1.484815179 | -1.268622988 |
| SST | -1.835290759 | -1.499521006 |
| TFAP2A | -2.367339503 | -1.826434342 |
| TFAP2A | -2.367339503 | -1.796460918 |
| UCP2 | 1.409043271 | 1.462641205 |
| UPP1 | -1.280808019 | -1.298120582 |

**Supplementary Table 3. Significantly altered biological pathways identified in PEO4 cells expressing *SHMT1* shRNAs compared to the cells expressing nonsilencing shRNA**

| S.No. | Enriched pathway map | p-value | FDR | In data | Network objects from active data |
|---|---|---|---|---|---|
| 1 | Immune response HMGB1/TLR signaling pathway | 1.339E-04 | 1.206E-02 | 3 | IL-6, IL-8, TIRAP (Mal) |
| 2 | Immune response Bacterial infections in normal airway | 3.373E-04 | 1.206E-02 | 3 | IL-6, IL-8, TIRAP (Mal) |
| 3 | PDE4 regulation of cyto/chemokine expression in arthritis | 3.373E-04 | 1.206E-02 | 3 | PDE4, IL-6, IL-8 |
| 4 | PDE4 regulation of cyto/chemokine expression in inflammatory skin diseases | 3.582E-04 | 1.206E-02 | 3 | PDE4, IL-6, IL-8 |
| 5 | Immune response_HSP60 and HSP70/ TLR signaling pathway | 4.499E-04 | 1.206E-02 | 3 | IL-6, IL-8, TIRAP (Mal) |
| 6 | Immune response_TLR2 and TLR4 signaling pathways | 5.277E-04 | 1.206E-02 | 3 | IL-6, IL-8, TIRAP (Mal) |
| 7 | Immune response_Role of PKR in stress-induced antiviral cell response | 5.277E-04 | 1.206E-02 | 3 | IL-6, IL-8, TIRAP (Mal) |
| 8 | Multiple myeloma (general schema) | 1.140E-03 | 2.279E-02 | 2 | IL-6, FGFR3 |
| 9 | Immune response_MIF-mediated glucocorticoid regulation | 1.709E-03 | 3.038E-02 | 2 | IL-6, IL-8 |
| 10 | Immune response_Role of HMGB1 in dendritic cell maturation and migration | 2.574E-03 | 4.025E-02 | 2 | IL-6, IL-8 |
| 11 | Immune response_Antigen presentation by MHC class I | 2.767E-03 | 4.025E-02 | 2 | CD8 alpha, CD8 |
| 12 | Colorectal cancer (general schema) | 3.174E-03 | 4.231E-02 | 2 | IL-6, IL-8 |
| 13 | Complement pathway disruption in thrombotic microangiopathy | 4.800E-03 | 4.604E-02 | 2 | IL-8, Factor I |
| 14 | Immune response_Role of integrins in NK cells cytotoxicity | 5.058E-03 | 4.604E-02 | 2 | IL-8, ICAM2 |
| 15 | Substance P-mediated inflammation and pain in Sickle cell disease | 5.058E-03 | 4.604E-02 2 | 2 | IL-6, IL-8 |
| 16 | Immune response_TSLP signalling | 5.322E-03 | 42.604E-02 | 2 | IL-6, IL-8 |
| 17 | Immune response_MIF in innate immunity response | 5.593E-03 | 4.604E-02 | 2 | IL-6, IL-8 |
| 18 | Immune response_Substance P-stimulated expression of proinflammatory cytokines via MAPKs | 6.441E-03 | 4.604E-02 | 2 | IL-6, IL-8 |
| 19 | Role of platelets in the initiation of in-stent restenosis | 6.441E-03 | 4.604E-02 | 2 | IL-8, ICAM2 |
| 20 | Immune response_IL-1 signaling pathway | 6.736E-03 | 4.604E-02 | 2 | IL-6, IL-8 |
| 21 | Immune response_PGE2 signaling in immune response | 7.038E-03 | 4.604E-02 | 2 | PGES, IL-8 |
| 22 | Immune response_Inhibitory action of Lipoxins on pro-inflammatory TNF-alpha | 7.345E-03 | 4.604E-02 | 2 | IL-6, IL-8 |

| | | | | | |
|---|---|---|---|---|---|
| | signaling | | | | |
| 23 | Chemotaxis_CCR1 signaling | 7.977E-03 | 4.604E-02 | 2 | IL-6, IL-8 |
| 24 | Immune response_C3a signaling | 7.977E-03 | 4.604E-02 | 2 | IL-6, IL-8 |
| 25 | Immune response_Histamine H1 receptor signaling in immune response | 7.977E-03 | 4.604E-02 | 2 | IL-6, IL-8 |
| 26 | Immune response_TLR5, TLR7, TLR8 and TLR9 signaling pathways | 7.977E-03 | 4.604E-02 | 2 | IL-6, IL-8 |
| 27 | Role and regulation of Prostaglandin E2 in gastric cancer | 8.302E-03 | 4.604E-02 | 2 | PGES, IL-8 |
| 28 | Development_PEDF signaling | 8.302E-03 | 4.604E-02 | 2 | IL-6, BDNF |
| 29 | Huntington's disease (general schema) | 8.443E-03 | 4.604E-02 | 2 | BDNF |
| 30 | Immune response_Histamine signaling in dendritic cells | 8.633E-03 | 4.604E-02 | 2 | IL-6, IL-8 |
| 31 | Inflammatory factors-induced expression of mucins in normal and asthmatic epithelium | 8.970E-03 | 4.630E-02 | 2 | IL-6, IL-8 |
| 32 | Immune response_Inhibitory PD-1 signaling in T cells | 9.661E-03 | 4.684E-02 | 2 | CD8 alpha, CD8 |
| 33 | Immune response_HMGB1/RAGE signaling pathway | 9.661E-03 | 4.684E-02 | 2 | IL-6, IL-8 |
| 34 | Th17 cells in CF | 1.002E-02 | 4.713E-02 | 2 | IL-6, IL-8 |

**Supplementary Table 4. Comparative mRNA (Common genes) fold change between SHMT1 Knockdown and CMAS Knockdown (Kohnz et.al., 2016 PMID: 27380425)**

| Gene Symbol | Fold Change- SHMT1 shRNA#1 | Fold Change- SHMT1 shRNA#2 | Fold Change- (CMAS knockdown) |
|---|---|---|---|
| KRT81 | -1.8 | -1.9 | -0.920299329 |
| SLC2A3 | -2.6 | -1.4 | -0.5326393 |
| BDNF | -1.6 | -1.3 | -0.691482534 |
| IL6 | -1.5 | -1.3 | -0.30881392 |
| KANK4 | -1.5 | -1.6 | -0.93313234 |
| CFI | -1.5 | -1.4 | -0.81327496 |
| ADM | -1.5 | -1.4 | -0.845720677 |
| NT5E | -1.5 | -1.6 | -0.558947094 |
| SRPX2 | -1.5 | -1.3 | -0.840808638 |
| PDE4B | -1.4 | -1.4 | -0.99759763 |
| KRT17 | -1.4 | -1.3 | -0.726446341 |
| CXCL8 (IL8) | -1.3 | -2.2 | -0.744391055 |
| UPP1 | -1.3 | -1.3 | -0.793003389 |
| ATOH8 | 1.3 | 1.7 | 1.353736295 |
| FAM83D | 1.4 | 1.3 | 1.263891559 |
| FGFR3 | 1.5 | 1.4 | 1.199907978 |
| DCAKD | 1.5 | 1.3 | 1.036156948 |
| RASD1 | 1.7 | 1.4 | 2.45188495 |

**Supplementary Table 5:** Primer sequences for RT-qPCR analysis; clone ID and catalog numbers for shRNAs (Open Biosystems); antibodies used; source and concentration of chemical inhibitors used- one

| **Application** | **Gene symbol** | **Forward primer (5′-3′)** | **Reverse primer (5′-3′)** |
|---|---|---|---|
| RT-qPCR | *SHMT1* | TCCCGAGGACTTTTGGAAGA | TGCCACAGCACTCTGGATCT |
| | *SHMT2* | GCTCAACCTGGCACTGACTG | CACTGATGTGGGCCATGTCT |
| | *DHFR* | TGAGGGATGATGGTTGCTTG | TGCCTGGCTGGTTTTTCTTT |
| | *GGH* | GCACATGCCTTGGATTTGAA | CGGAGAGGCTCCACTTATGG |
| | *PUR9 (ATIC)* | GTGTCCACGGAGATGCAGAG | AGCTTGGGCTGCAGTGTGTA |
| | PUR2 (GART) | TTTGGTTGTGAAGGCCAGTG | TCCCATTCCCCCTGTGTTAG |
| | *WT1* | AGCAGGAAGCACACTGGTGA | ACCGGGCAAACTTTTTCTGA |
| | *IL6* | TGTAACCGCCCCACACAGAC | TTCTGCCAGTGCCTCTTTGC |
| | *IL8* | TAAAAAGCCACCGGAGCACT | ATCAGGAAGGCTGCCAAGAG |
| CHIP assay | | | |
| | WT1 Promoter *SHMT1* ChIP | GAGAAACAGGCCCTGCCC | GAACTTGGCGCTGGACCG |
| | | | |
| | **Gene symbol** | **Clone ID** | **Catalog number** |
| shRNA | *SHMT1* | RHS3979-9602172 | TRCN0000034764 |
| | | RHS3979-9602173 | TRCN0000034765 |
| | *SHMT2* | RHS3979-9602213 | TRCN0000034805 |
| | | RHS3979-9602214 | TRCN0000034806 |
| | *DHFR* | RHS3979-9606408 | TRCN0000039000 |
| | | RHS3979-9606411 | TRCN0000039003 |
| | *GGH* | RHS3979-9618537 | TRCN0000051338 |
| | | RHS3979-9618541 | TRCN0000051342 |
| | PUR9 (ATIC) | RHS3979-9602372 | TRCN0000034964 |
| | | RHS3979-9602375 | TRCN0000034967 |
| | PUR2 (GART) | RHS3979-9602315 | TRCN0000034907 |
| | | RHS3979-9602316 | TRCN0000034908 |
| | *WT1* | RHS3979-9569562 | TRCN0000001114 |
| | | RHS3979-9569563 | TRCN0000010596 |
| | | | |
| Immunoblotting / CHIP | **Protein symbol** | **Antibody source** | **Dilution** |
| | *SHMT1* | Cell signaling | 1:5000 |
| | *WT1* | Cell signaling | 1:5000 |
| | Actin | Cell signaling | 1:5000 |
| | IL6 | Santa cruz | 1:500 |
| | IL8 | Santa cruz | 1:500 |
| | | | |
| | **Compounds** | **Concentration** | **Source** |
| | P-3Fax-Neu5Ac | Different concentrations as indicated | Tocris |
| | Neu5Ac | Different concentrations as indicated | Santa Cruz |
